# Rewiring cattle movements to limit infection spread

**DOI:** 10.1186/s13567-024-01365-z

**Published:** 2024-09-19

**Authors:** Thibaut Morel-Journel, Pauline Ezanno, Elisabeta Vergu

**Affiliations:** 1 Oniris, INRAE, BIOEPAR, 44300 Nantes, France; 2 Université Paris-Saclay, INRAE, MaIAGE, 78350 Jouy-en-Josas, France

**Keywords:** Control strategy, epidemiology, data-based, network, stochastic model

## Abstract

Cattle tracing databases have become major resources for representing demographic processes of livestock and assessing potential risk of infections spreading by trade. The herds registered in these databases are nodes of a network of commercial movements, which can be altered to lower the risk of disease transmission. In this study, we develop an algorithm aimed at reducing the number of infected animals and herds, by rewiring specific movements responsible for trade flows from high- to low-prevalence herds. The algorithm is coupled with a generic computational model based on the French cattle movement tracing database (BDNI), and used to describe different scenarios for the spread of infection within and between herds from a recent outbreak (epidemic) or a five-year-old outbreak (endemic). Results show that rewiring successfully contains infections to a limited number of herds, especially if the outbreak is recent and the estimation of disease prevalence frequent, while the respective impact of the parameters of the algorithm depend on the infection parameters. Allowing any animal movement from high to low-prevalence herds reduces the effectiveness of the algorithm in epidemic settings, while frequent and fine-grained prevalence assessments improve the impact of the algorithm in endemic settings. Our approach focusing on a few commercial movements is expected to lead to substantial improvements in the control of a targeted disease, although changes in the network structure should be monitored for potential vulnerabilities to other diseases. This general algorithm could be applied to any network of controlled individual movements liable to spread disease.

**Supplementary Information:**

The online version contains supplementary material available at 10.1186/s13567-024-01365-z.

## Introduction

Following bovine spongiform encephalopathy and classical swine fever epidemics in the 1990s, the European Union initiated the mandatory identification and registration of cattle [1]. This decision led to the creation of national identification databases, such as the cattle tracing system in the United Kingdom [2, 3], the French national bovine identification database (BDNI) [4, 5], the Italian national bovine database [6, 7] and the database of the Swedish board of agriculture [8, 9]. Of course, such databases also exist outside Europe, for instance in West Africa [10], Chile [11], Paraguay [12] or Brazil [13]. These animal tracing systems have enabled the monitoring of infectious livestock diseases and the development of strategies to prevent their spread [14–16], since animal trade is a major transmission pathway between herds. Indeed, commercial exchanges are not only recorded comprehensively, but also controlled by farmers, unlike animal mobility in the wild. These databases, whose reliability has increased over time since their creation [17], are therefore powerful tools for simulating infectious diseases in cattle [13, 18, 19] and assessing the impact of livestock movements on epidemics [11, 12, 20].

The information provided by these commercial animal movements can be used as a basis for representing comprehensively the demographic processes and trades between cattle farms located in a given region, using a metapopulation framework [21, 22]. To this end, disease transmission between individuals within a defined set of herds can be modelled, by combining an epidemiological model with existing data on births, deaths and movements. This type of models accounts at least for two ways of spreading the infection: by contact within a herd, or by actually moving animals between herds. This is for instance the case for paratuberculosis, a cattle disease mainly spread between herds by trade [23, 24]. Manipulating the structure of cattle movement is expected to have a direct impact on the latter and an indirect impact on the former.

The structure of these trade movements can be understood through the prism of graph theory: herds are the vertices of a commercial exchange network, whose edges are the movements of livestock [25]. Thus, each herd can be characterised using graph metrics, e.g. the in- and out-degree, i.e. the number of herds it has respectively bought animals from and sold animals to. Network-based control strategies then aim to modify the structure of the network to reduce infection risks. Removing vertices [4, 26] or edges [27, 28] through trade ban or culling is a method used to slow down epidemics. In a context of cattle exchange however, preventing farmers from buying or selling livestock entails high economic costs. Therefore, this strategy cannot be used routinely or over extended periods of time. It is likely better suited to the management of regulated diseases, the consequences of which are also very costly and for controlling outbreaks of newly introduced diseases. Conversely, the application of such drastic methods on the longer term for endemic diseases may not be feasible.

Edge rewiring is a less radical approach able to balance the trade-off between health risks and economic costs. This method corresponds to the modification of one or both vertices that an edge connects [29–32]. Although most of the theoretical literature on the subject rather considers rewiring in the context of human contact networks, it has also been used to study epidemic spread in cattle movement networks [20, 33–35]. For instance, Gates and Woolhouse [33] present a rewiring method that creates an entirely new movement network disconnecting large buyers from large sellers, while retaining the total number of animals bought or sold by each herd. This method requires information at the network level, the criteria used being the distributions of in- and out-degrees of all herds. Global-level information is also generally required for most rewiring methods in contact networks, although Piankoranee and Limkumnerd [30] proposed a method based on local information. In their study, rewiring is decided at the vertex level, according to its status and those of its direct neighbours. Controlling cattle movements depending on the sanitary status of their origin has been proposed in previous studies, e.g. by Hidano et al. [36]. Their study presents different scenarios regarding farmers’ practices, especially their tendency to avoid buying cattle from regions with a higher incidence of bovine paratuberculosis. The approach presented here is similar, albeit at a finer grain: preventing farmers from buying cattle from herds with a higher prevalence of the target disease.

This study presents a new rewiring method to reduce the spread of infections in a cattle movement network. To do this, we developed a rewiring algorithm aimed at preventing the movements of animals from higher-prevalence herds to lower-prevalence ones. It was based on an edge-level criterion: the estimated difference in prevalence between the herd of origin and the herd of destination of the movement considered. For this study, we tested the algorithm in conjunction with a computational epidemiological model describing the spread of a nonspecific disease, whose infectiousness was parametrically defined. The impact of the algorithm was tested using a real commercial movement network, based on dataset from the French cattle tracing system (BDNI). In contrast with similar rewiring approaches developed recently to target specific diseases [20, 35], we propose a more generalist approach aimed at investigating the effectiveness of this type of method in a broader context. After presenting the movement network used as an example, the model and the algorithm, we consider various outputs of simulations with and without rewiring, concerning the functioning of the algorithm itself, its impact on infection propagation, and on the structure of the cattle movement network.

## Materials and methods

### Cattle movement network

In order to test the algorithm on an actual network of commercial bovine movements, we use an extraction from the French national bovine identification database (BDNI). It includes all cattle herds in Brittany (a French region) that sold or bought at least one animal during the year 2014. This set of 21 548 herds is referred to as the “metapopulation” thereafter. Every animal in the dataset is included regardless of breed or age. Three types of commercial exchanges are considered: (i) “internal movements” have an origin and a destination among the herds in the dataset, (ii) “imports” have only a destination in the dataset and (iii) “exports” have only an origin in the dataset. They represent respectively 64%, 16% and 20% of the commercial exchanges involving at least one herd of the metapopulation. Each commercial exchange of animals is assumed to take place directly from one herd to another, neglecting intermediaries. Hence, markets and sorting centres are not considered for this study. They differ from herds in that they tend to concentrate a large number of animals, but for a limited period of time (less than a day for markets, a few days for sorting centres). Besides, the dataset also includes information about demographic events, which are considered as a special type of movement: (iv) births have only a destination, corresponding to the herd where the animal is born, and (v) deaths are considered the same way as exports: they have only an origin, corresponding to the last herd recorded for the animal.

The dataset is represented as a network, with herds and internal movements corresponding to the vertices and edges, respectively. This network is (i) dynamic, i.e. movements are characterised by the date at which they occur, (ii) weighted, i.e. a single edge represents the set of all movements from herd *A* to herd *B*, with a weight corresponding to the number of movements, and (iii) directed, i.e. movements from herd *A* to herd *B* are accounted for separately from movements from herd *B* to herd *A*. This network includes 21,548 vertices and 100 088 edges. The total number of internal movements over the year 2014 is 206 640, so the average edge weight is 2.06.

### Epidemiological model: within and between-herd dynamics and epidemiological settings

The model developed aims to simulate pathogen transmission within herds, and infection spread between herds through cattle movements. A full description of the model is included in Additional file 1. The model is stochastic in discrete time—each time-step corresponding to a day of 2014—and in discrete space—by integrating the network of herds and movements described above. Commercial exchanges and demography are data-based: movement *m* is characterised by its origin *O*_*m*_, its destination *D*_*m*_, its date according to the dataset *T*_*m*_^∗^ and the date at which it is simulated *T*_*m*_. By default, movements are simulated according to the dataset, i.e. *T*_*m*_ = *T*_*m*_^∗^. Within-herd dynamics are based on a SIRS model with three parameters: the infection rate *β*, the recovery rate *γ*—therefore the average infection duration is 1*/γ*—and the rate of return to susceptibility *δ*. Transmission is here modelled as density- dependent, i.e. contact rates are assumed to increase with the number of animals in the herd, as opposed to frequency-dependent transmission, i.e. contacts are assumed to be constant. Both methods are used to model cattle diseases [37–39], and there is no strong incentive to choose one over the other for this study, as the epidemiological model does not aim at reproducing a specific disease. The surface area of holdings can affect the density-dependent transmission rate in the herds, so that contacts actually depend on the ratio between numbers and area [38, 40]. However, this area cannot be easily inferred from the number of animals, as the relationship between the two is not necessarily proportional and might depend on cattle management practices (e.g. extensive vs. intensive). Since these surface areas are not known, they are simply assumed to be the same. It should be noted that this choice could lead to disproportionately high modelled transmission rates in the largest herds. At each time-step *t*, herd *h* is characterised by its number of susceptible, infected and recovered individuals, noted respectively *S*_*h*_(*t*), *I*_*h*_(*t*) and *R*_*h*_(*t*). The total herd size *N*_*h*_(*t*) is defined as the sum of these three values and infection prevalence as *P*_*h*_(*t*) = *I*_*h*_(*t*)*/N*_*h*_(*t*).

Each simulated infection begins with an initial outbreak in a metapopulation without infection, i.e. with only susceptible individuals. At *t* = *t*_*I*_, the date of the outbreak, 10% of all herds in the metapopulation are simultaneously infected, by replacing 1 susceptible individual with 1 infected individual in each of the herds. The initially infected herds are randomly drawn for each simulation, so that a variety of potential initial outbreaks is tested. The probability that a herd is initially infected is proportional to the number of imports in the herd, according to the 2014 dataset. The rationale is that herds receiving the most individuals from herds outside of the metapopulation are the most likely to introduce a new infection.

Two types of infections are considered for the study: epidemic and endemic. An infection is defined as “epidemic” if its outbreak occurs at the start of the simulation, i.e. if *t*_0_ = *t*_*I*_. The initial state of the infection is then as described above. An infection is defined as “endemic” if its outbreak occurs five years before the start of the simulation, i.e. *t*_0_ = *t*_*I*_ + 1825 days. In this case, the initial state of infection is the result of preliminary simulation of a five-year infection using the same epidemiological model and a cattle movement network also retrieved from the BDNI, similar to the 2014 dataset presented above, but concerning herds exchanging cattle in Brittany from 2009 to 2013. This five-year movement network includes 26 075 different herds exchanging cattle (20 367 herds per year on average), for a total of 1 061 580 movements (212 316 movement per year on average). Preliminary simulations for which the infection goes extinct before *t*_0_ are discarded, so that no disease-free initial state is considered.

Six epidemiological settings are defined, corresponding to three sets of parameters of the SIRS model (*β*, *γ* and *δ*) for each of the two infection types (epidemic or endemic). Additional file 2 presents two clustering analyses performed on simulations of the model over the year 2014 to define these infection settings. For both endemic and epidemic diseases, this analysis discriminates between three settings that correspond to the overall severity of the disease: weak, moderate or strong. The parameters values of the SIRS model corresponding to a given epidemiological setting are the average values of the simulations belonging to the corresponding cluster.

### Prevalence status of the herds

The algorithm developed for this study performs rewiring based on herd prevalence classes, numbered from 1 to *c*. Class *i* includes all prevalence values between *b*_*i*_ and *b*_*i*+1_, with the lowest boundary *b*_1_ = 0 and the highest boundary *b*_*c*+1_ = 1. At time *t*, herd *h* is assigned a “real” prevalence status, noted *V *^*r*^(*t*), corresponding to the prevalence class including its prevalence, i.e. *V*_*h*_^*r*^(*t*) = *i* if *P*_*h*_(*t*) ∈ [*b*_*i*_; *b*_*i*+1_[, with *V*_*h*_^*r*^(*t*) = *c* + 1 if *P*_*h*_(*t*) = 1. Yet, the algorithm does not actually use this status, but rather an ‘observed’ prevalence status, noted *V*_*h*_^*o*^(*t*). The observed status is equal to the real status recorded at time *t*_*obs*_, which then remains the same for *q* time-steps, i.e. *V*_*h*_^*o*^(*t*) = *V*_*h*_^*r*^(*t*_*obs*_) ∀ *t* ∈ [*t*_*obs*_; *t*_*obs*_ + *q*[. At the next recording *q* time-steps later, i.e. at *t*_*obs*_ + *q*, the observed status of the herd is updated to correspond to the real status at that date. No additional error on the observed status is assumed, so that it always corresponds to the real status at *t*_*obs*_. Additional file 3 presents an assessment of the impact of other sources of error, such as imperfect test sensitivity or testing a subset of animals in the herd, on the accuracy of herd statuses. Conversely to differences between the real (*V*_*h*_^*r*^(*t*)) and observed prevalence status (*V*_*h*_^*o*^(*t*)), which accumulate over time since the last observation, such sources of errors generate an additional uncertainty about observed status constant over time. Both sources of error contribute in a similar way to this uncertainty, but the number of herds assigned the right status is greater than 70% for the most extreme case tested (only 30% of animals tested with a sensitivity of 0.5 for a strong disease), and remains above 80% when considering weak or moderate epidemiological settings. While their impact can become notable in these worst cases, classes therefore remain a valid proxy for the prevalence of the herds, which is used here to evaluate the commercial exchanges between herds. More precisely, the algorithm aims at identifying and preventing movements “at risk”, i.e. for which the observed status of the origin is strictly greater than that of the destination.

### Sequential rewiring

The algorithm works by permuting the origins of pairs of movements, one of which is at risk, so that neither of them is at risk after the rewiring. The pairs of movements are created such that 1 ≤ *c*_*ON*_ ≤ *c*_*DR*_ < *c*_*OR*_ ≤ *c*_*DN*_ ≤ *c*, with *c*_*OR*_ and *c*_*DR*_ the observed status of the origin and destination of the movement at risk, while *c*_*ON*_ and *c*_*DN*_ are the observed status of the origin and destination of the other movement. By permuting the origins, the algorithm creates a movement with an origin of status *c*_*ON*_ and a destination of status *c*_*DR*_, and another movement with an origin of status *c*_*OR*_ and a destination of status *c*_*DN*_. Then, neither of the two movements is at risk, since *c*_*ON*_ ≤ *c*_*DR*_ and *c*_*OR*_ ≤ *c*_*DN*_.

For all movements set to occur at a given time-step, the algorithm performs these permutations in a specific order to ensure that the algorithm performs every possible rewiring. Additional file 4 describes in pseudo-code the functioning of the algorithm over a single time-step, which proceeds as follows. Firstly, it defines all possible quadruplets of prevalence classes {*c*_*OR*_, *c*_*DR*_, *c*_*ON*_, *c*_*DN*_}. These quadruplets are arranged primarily in ascending order of *c*_*DR*_, secondarily in descending order of *c*_*OR*_, thirdly in ascending order of *c*_*ON*_ and fourthly in descending order of *c*_*DN*_, which ensures that no potential permutation is missed. For each quadruplet, the algorithm then permutes the origins of *k* pairs of movements, with *k* the minimum between the number of movements at risk and the number of other movements considered.

Once all possible permutations are performed, there might be remaining movements at risk set to be performed on this time-step. Firstly, these remaining movements are postponed to the next day, to be potentially rewired with another set of movements. The postponed movements are then prioritised for rewiring on the following day. Yet, postponing commercial movement represents a constrain for farmers. Therefore, a maximal delay during which a movement can be postponed ∆_*MAX*_ is fixed for the algorithm. Thus, remaining movement *m* is postponed to the next day only if it was not already postponed ∆_*MAX*_ days, i.e. if *T*_*m*_ − *T*_*m*_^∗^  < ∆_*MAX*_. If the algorithm prohibits any movement at risk, the remaining movements that cannot be postponed (called ‘problematic’ movements) are replaced by one export with the origin of the problematic movement as origin and one import with the destination of the problematic movement as destination. If the algorithm does not prohibit any movement at risk, the problematic movement is set to occur as such. Overall, the algorithm therefore depends on four parameters: the number of prevalence classes *c*, the period at which observed status is updated *q*, the maximum delay ∆_*MAX*_ and whether movements at risk are prohibited.

### Simulations

Simulations of infections with or without using the algorithm are performed on the dataset between 01/01/2014 (defined as *t* = 0) and 01/01/2015 (*t* = 365). The effectiveness of the algorithm is tested by running simulations with 3 × 3 × 3 × 2 combinations of the algorithm parameters, respectively (i) the number of prevalence classes *c* (2, 3 or 4 classes), (ii) the update period *q* (1, 28 or 91 days), the maximum delay ∆_*MAX*_ (1, 3 or 7 days) and (iv) the prohibition of movements at risk (yes or no). Each combination, as well as a control without rewiring, is simulated 100 times for each of the six epidemiological settings defined above.

As indicated above, preliminary simulations are carried out for each epidemiological setting between 01/01/2009 (*t* =  − 1825) and 31/12/2013 (*t* =  − 1), with an initial outbreak at *t*_*I*_ =  − 1825. The distributions of prevalence values in the metapopulation at *t* =  − 1 are used to define the boundaries of the prevalence classes used by the algorithm. To account for the potentially large number of disease-free herds, two methods are defined. If less than 1*/c* herds are disease-free, *b*_*i*_ is defined as the ((*i* − 1)*/c*)th quantile of the distribution. This is the case for *c* = 2 in the strong epidemic and endemic epidemiological settings (Additional file 5). If more than 1*/c* herds are disease-free, the first class includes only disease- free herds, i.e. *b*_1_ = *b*_2_ = 0, while *b*_*i*_ is the ((*i* − 2)*/*(*c* − 1))th quantile of the distribution for *i* > 2. This is the case for all the other combinations of epidemiological settings and values of *c* (Additional file 5), so that the algorithm includes a disease-free class in most simulations.

Using quantiles to define classes ensures that herds are distributed equally between them. Hence, the actual boundary values increase with the severity of the disease, being lower for weak epidemiological scenarios for which prevalence levels are expected to be overall lower, and higher for strong epidemiological scenarios for which they are expected to be overall higher (Additional file 5). Considering the same boundaries between classes across epidemiological scenarios could lead to very unevenly distributed herds across prevalence classes, making some classes essentially useless for rewiring.

### Outcomes and analyses of numerical explorations

The simulations outcomes are listed in Table 1. They are related either to (i) the functioning of the algorithm, (ii) the infection or (iii) the network of internal movements modified by the algorithm.

**Table 1:** **Outcomes computed from the simulations**

Outcomes related to	Notation	Description
Algorithm	*n*_*rew*_(*t*)	Number of movements rewired at time *t*
	*n*_*del*_(*t*)	Number of delayed movements at time *t*
	*n*_*prob*_(*t*)	Number of problematic movements at time *t*
	*n*_*risk*_(*t*)	Number of movements at risk at time *t*
	*n*_*err*_(*t*)	Number of movements undetected as at risk at time *t*
Infection	*n*_*inf*_	Number of herd infections
	*next*	Number of herds in which the infection goes extinct
	*adur*	Average duration of infection
	*c*_*inc*_(*t*)	Cumulative incidence at time *t*
	*n*_*herd*_(*t*)	Number of infected herds at time *t*
	*n*_*ind*_(*t*)	Number of infected individuals in the metapopulation at time *t*
	*a*_*prev*_(*t*)	Average prevalence in the infected herds at time *t*
Network	*n*_*SCC*_	Number of strongly connected components
	*max*_*SCC*_	Size of the largest strongly connected component
	*ind*_*h*_	In-degree of herd *h*
	*outd*_*h*_	Out-degree of herd *h*

Outcomes of the simulations related to the behaviour of the algorithm, the spread of the simulated infection or the structure of the movement network. The infection-related outcomes were computed for each simulation separately. The algorithm and network-related ones were computed for each simulation with the algorithm.

The algorithm-related outcomes *n*_*rew*_(*t*), *n*_*del*_(*t*) and *n*_*prob*_(*t*) are computed each time-step after rewiring, while *n*_*risk*_(*t*) and *n*_*err*_(*t*) are computed before rewiring. These latter outcomes are computed by using the real prevalence status of the herds, rather than the observed ones. A movement *m* is included in *n*_*risk*_(*t*) if *V*_*Om*_^*r*^(*t*) > *V*_*Dm*_^*r*^(*t*), and also included in *n*_*err*_(*t*) if *V*_*Om*_^*o*^(*t*) ≤ *V*_*Dm*_^*o*^(*t*) at the same time. The proportion of undetected movements at risk is computed on a weekly basis, to account for intra-week variability in the number of livestock movements. Over week w, this proportion *p*_*err*_(*w*) is:

$$p_{err} \left( w \right) = \frac{{\mathop \sum \nolimits_{{t = 7\left( {w - 1} \right) + 1}}^{7w} n_{err} \left( t \right)}}{{\mathop \sum \nolimits_{{t = 7\left( {w - 1} \right) + 1}}^{7w} n_{risk} \left( t \right)}}$$

The Spearman’s correlation coefficient *ρ* between *p*_*err*_(*w*) and the number of weeks since last update (from 1 to 4 weeks if *q* = 28 days, from 1 to 13 weeks if *q* = 91 days) is also computed to assess the relationship between errors in herd prevalence status and time. The Spearman’s coefficient is preferred because it does not assume any particular distribution of the involved variables.

The impact of the algorithm on the infection dynamic is estimated through *c*_*inc*_(*t*), i.e. the cumulative number of herds newly infected over the simulation. The variations in *n*_*herd*_(*t*) and *n*_*ind*_(*t*) over time are also presented in Additional file 6. Besides, the overall impact of the algorithm on the infection is assessed using a global multivariate sensitivity analysis, following Lamboni et al. [41] and using the *multisensi* package of the *R* software [42], which is used to perform sensitivity analyses on a multivariate output. For this analysis, twelve variables are derived from the infection-related outcomes. The three outcomes computed once per simulation *n*_*inf*_, *n*_*ext*_ and *a*_*dur*_ are used as such. In addition, the maximum, minimum and final values over the whole period simulated (respectively noted *max*(*u*(*t*)), *min*(*u*(*t*)) and *u*(365) for outcome *u*(*t*)) of *n*_*herd*_(*t*), *n*_*ind*_(*t*) and *a*_*prev*_(*t*) are also computed. The analysis includes a principal component analysis (PCA) on the scaled variables, which are used as the multivariate output for the sensitivity analysis. Two generalised sensitivity indices (GSI), which are weighted means of the sensitivity indices over all the dimensions of the PCA, are computed for each algorithm parameter: the total index (tGSI) including interactions with other parameters, and the first-order index (mGSI), not including them. The first principal component of the PCA is also used to assess the distribution of the simulations depending on the algorithm parameters.

The network-related outcomes are based on a static view of the network aggregating all the internal movements performed during the simulation, from *t* = 0 to *t* = 365. Therefore, they take into account the rewiring performed by the algorithm, and the potential removal of problematic movements if movements at risks are completely prohibited. The outcomes recorded for the modified networks are compared to the same metrics for the original network defined by the 2014 dataset. The strongly connected components—from which *n*_*SCC*_ and *max*_*SCC*_ are computed—correspond to groups of vertices linked to each other by a directed path. The percentiles of the distributions of *ind*_*h*_ and *outd*_*h*_ of all herds in the static network are used to assess the in-degree and out-degree distributions, respectively.

## Results

### Outcomes related to the algorithm

Our results show that number of movements rewired varies greatly depending on the date of the outbreak. It is negligible in the epidemic settings, with 80% of simulations with a total of rewired movements between 192 (fewer than 0.1% of all movements) and 2250 (1.1%). However, it is larger in the endemic settings, with 80% of simulations with between 17 344 (8.4% of all movements) and 33 640 (16.3%) movements rewired. Besides, increasing the value of ∆_*MAX*_ logically increases the number of delayed movements (which is 0 by definition for ∆_*MAX*_ = 0) and decreases the number of problematic movements. In the endemic settings, the problematic movements represent a small proportion of the movements detected as high risk (median: 5.4%, 9^th^ decile: 17.4%). In the epidemic settings however, they represent a larger part (median: 14.3%, 9^th^ decile: 59.7%), although their absolute numbers remain low (median: 129, 9^th^ decile: 651). Because of the overwhelming number of initially non-infected herds in these simulations, the movements at risk are likely more difficult to rewire, and thus more likely to be tagged as problematic by the algorithm.

**Figure 1:**
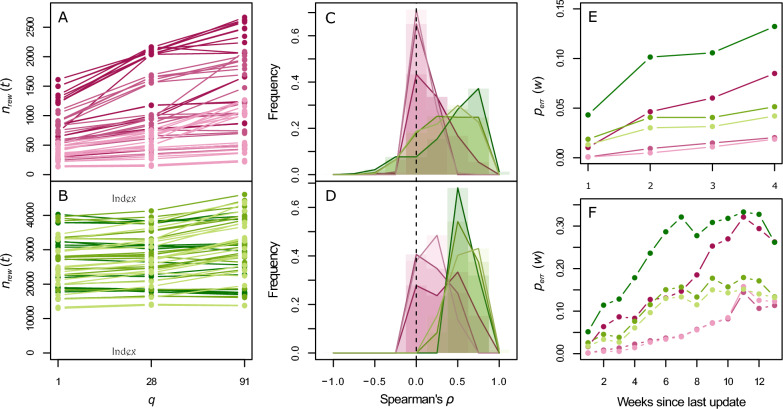
**Impact of the update period on the undetected movements at risk.** Impact of the update period *q* on the undetected movements at risk, in epidemic (magenta) or endemic settings (green), weak (light), moderate (medium) or strong (dark). First column: total number of rewiring events as a function of the update frequency *q*, averaged over all simulations for a same algorithm parameter combination, in epidemic (**A**) and endemic settings (**B**). Second column: distribution of Spearman’s correlation coefficients (*ρ*), with *q* = 28 days (**C**) and *q* = 91 days (**D**). Third column: average proportion of undetected movements at risk *p*_*err*_(*w*) as a function of the number of weeks since the last update, with *q* = 28 days (**E**) and *q* = 91 days (**F**).

Increasing the herd status update period *q* is not associated with a decrease in the number of rewiring events (Figures 1A, B). The value of *q* is even rather positively correlated with the number of rewiring events in epidemic settings. This suggests that the algorithm performs more erroneous rewiring as *q* increases. This is confirmed by the distributions of Spearman’s correlation coefficient between *p*_*err*_(*w*) and the number of weeks since last update *ρ* with *q* = 91 days (Figure 1D), in epidemic settings (80% of values of *ρ* between −0.01 and 0.50) and in endemic settings (80% of values of *ρ* between 0.39 and 0.75). This is also somewhat the case with *q* = 28 days (Figure 1C), although the correlations are weaker, in endemic (80% of values of values between −0.09 and 0.79) as well as in epidemic settings (80% of values of values between −0.05 and 0.34).

The average proportions of undetected movements at risk *p*_*err*_(*w*) all tend to increase with the number of weeks since the last update *w* (Figures 1E, F). This increase is systematically greater for the largest value of *q*, up to *p*_*err*_(*w*) = 0*.*3. However, they also appear to reach a plateau after 10 weeks. This suggests that a further increase in the update period *q* would not strongly increase the proportion of undetected movements at risk. As for Spearman’s correlation coefficient *ρ*, the increase is greater in endemic settings than in epidemic settings.

Additional file 3 presents an assessment of the impact of other sources of error, such as imperfect test sensitivity or testing a subset of animals in the herd, on the accuracy of herd statuses. While differences between the real (*V*_*h*_^*r*^(*t*)) and observed prevalence status (*V*_*h*_^*o*^(*t*)) are expected to accumulate over time since the last observation, including such sources of errors generate an additional uncertainty about observed status constant over time. Both sources of error contribute in a similar way to this uncertainty, although the number of herds assigned the right status never goes below 70% in the worst case tested (only 30% of animals tested with a sensitivity of 0.5), and below 80% when considering weak or moderate epidemiological settings only.

### Outcomes related to the infection

**Figure 2:**
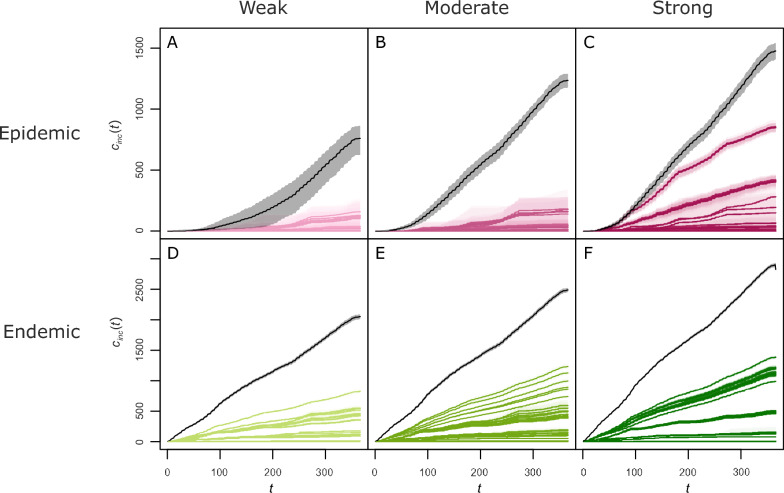
**Impact of the algorithm on the number of herd infections.** Cumulative incidence *c*_*inc*_(*t*), in number of herd infections, as a function of time (*t*, in days), for simulations with (colour) or without rewiring (black), in epidemic (1^st^ row, magenta) or endemic settings (2^nd^ row, green), weak (1^st^ column, light), moderate (2^nd^ column, medium) and strong (3^rd^ column, dark). Each combination of algorithm parameters is represented by its mean over the repetitions (solid line) and an interval of 80% of simulations (envelope).

Comparison of the results with and without rewiring shows the overall effectiveness of the algorithm in containing the infection (Figure 2). Regardless of the epidemiological setting and the combination of parameters considered, the cumulative number of herds newly infected *c*_*inc*_(*t*) remains systematically lower after rewiring. The algorithm is particularly effective in weak and moderate epidemic settings, where very few herds are infected during the year. In other epidemiological settings, the impact of the algorithm varies more strongly depending on the scenario considered. Results for *n*_*herd*_(*t*) and *n*_*ind*_(*t*) are presented in Additional file 6. In epidemic settings, variations in *n*_*herd*_(*t*) logically follow closely those of *c*_*inc*_(*t*). Hence, the algorithm also reduces the increase in the total number of infected herds. It also reduces the total number of infected individuals, although the impact is not as strong as for herds. In endemic settings, the value of *n*_*herd*_(*t*) remains similar during the whole simulation without rewiring (Additional file 6), despite new infections according to variations in *c*_*inc*_(*t*). This indicates a turnover in the infection at the metapopulation level, with populations losing the infection through the acquisition of resistance or the culling and trade of infected animals. By reducing the number of new infections, the algorithm therefore reduces the total number of infected herds over time. However, its impact is smaller on the total number of infected individuals (Additional file 6).

**Figure 3:**
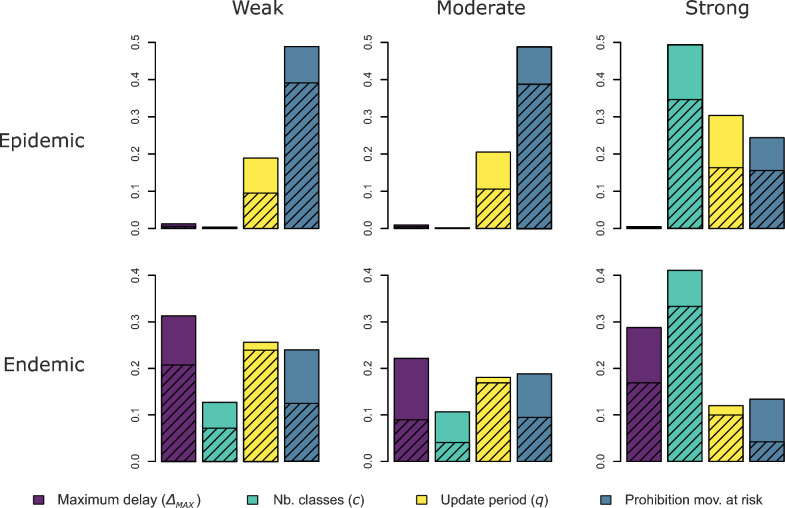
**Sensitivity analysis of the algorithm parameters.** Generalised sensitivity indices (GSI) of the maximum delay ∆_*MAX*_ (purple) the number of prevalence classes *c* (cyan), the update period *q* (yellow) and the prohibition of movements at risk (blue), in epidemic (1^st^ row) or endemic settings (2^nd^ row), weak (1^st^ column), moderate (2^nd^ column) and strong (3^rd^ column). The total indices (tGSI) are in solid colour and the first-order indices (mGSI) are hatched.

The sensitivity analysis shows differences in the relative importance of the algorithm parameters on the reduction of the infection (Figure 3). Three different patterns of sensitivity to the algorithm parameters are observed. Firstly, simulations in weak and moderate epidemic settings exhibit an overwhelming sensitivity to the prohibition of movements at risk. Secondly, those in strong epidemic or endemic settings exhibit a strong sensitivity to the number of prevalence classes *c*. Finally, those in weak and moderate endemic settings exhibit a more balanced sensitivity to all parameters, with a substantial difference between total and first-order indices for the maximum delay ∆_*MAX*_, the number of classes and the prohibition of movements at risk. These differences suggest an interaction between the three algorithm parameters. Besides, simulations for every epidemiological setting are somewhat sensitive to the update period *q*.

**Figure 4:**
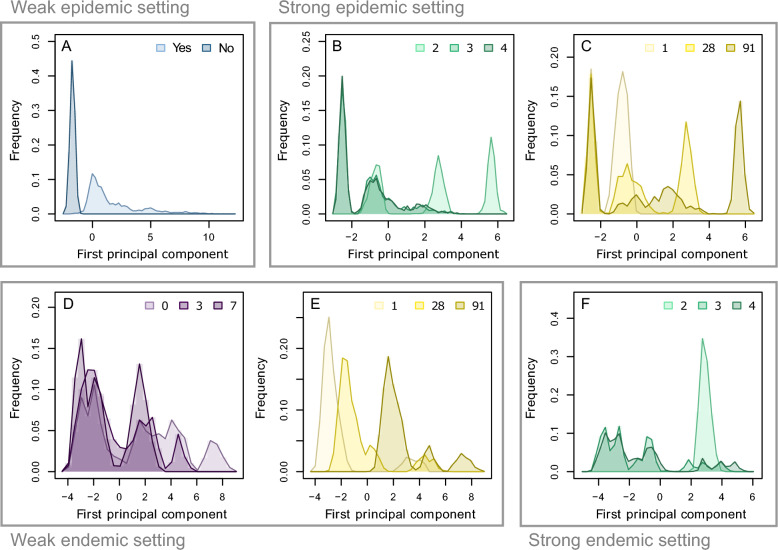
**Distribution of simulation outcomes depending on algorithm parameters.** Distribution of the simulations on the first component of the PCA performed as a first step of the sensitivity analysis, in the weak epidemic setting (**A**), the strong epidemic setting (**B**, **C**), the weak endemic setting (**D**, **E**) and the strong endemic setting (**F**). The outputs are divided by maximum delay (purple, **D**), management of problematic movements (blue, **A**), number of prevalence classes (cyan, **B** and **F**) and herd status update period (yellow, **C** and **E**).

The PCA performed as a first step of the sensitivity analysis is used to explore further the way algorithm parameters impact the infection-related outputs. Additional file 7 shows that the first principal component of the PCA is globally positively correlated with outputs describing the extent of the infection. The distributions of simulations along this first principal component therefore provides information about the way algorithm parameter values affects the extent of the infection. Additional file 8 presents these distributions for every epidemiological setting and every algorithm parameter, while Figure 4 displays some of the most relevant distributions. Figure 4A shows that, in the weak epidemic setting, simulations in which movements at risk are prohibited almost always score lower on the first principal component than those in which they are not. The distribution is similar in the moderate epidemic setting (Additional file 8), which has similar sensitivity indices (Figure 3). Interestingly, distributions of simulations in strong epidemic or endemic settings show that those with *c* = 2 score higher on their respective first component, while those with *c* = 3 and *c* = 4 are not different (Figures 4B, F). A similar pattern is observed with the maximum delay in the weak endemic setting: only simulations with ∆_*MAX*_ = 0 score higher on the first principal component (Figure 4D). In the strong epidemic setting, the two high-scoring peaks in the distribution according to *c* (Figure 4B) correspond to the simulations with *q* = 28 and *q* = 91 (Figure 4C), highlighting an interplay between the number of classes *c* and the update period *q*. No interplay between ∆_*MAX*_ and *q* is visible in the weak endemic setting, although Figure 4E shows that the score of simulations on the first principal component is positively correlated with *q*. Distributions in the moderate endemic setting are similar to those in the weak endemic setting (Additional file 8).

### Outcomes related to the movement network

**Figure 5:**
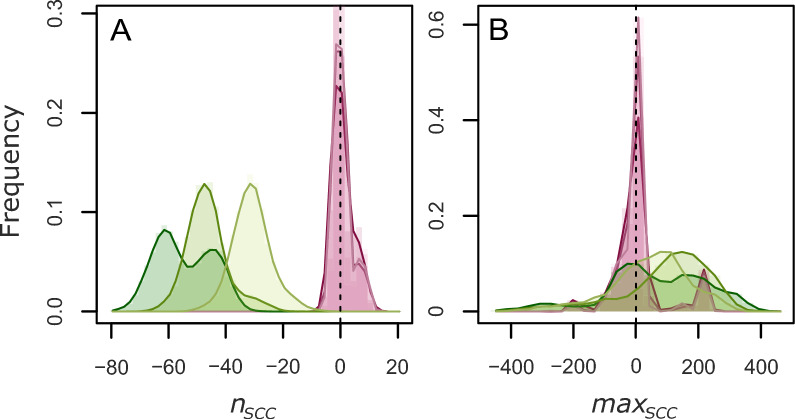
**Impact of the algorithm on the strongly connected components in the movement network.** Distributions of the differences in number of strongly connected components (**A**, *n*_*SCC*_) and in size of the largest strongly connected component (**B**, *max*_*SCC*_) between rewired networks and the original one from the dataset, for epidemic (magenta) and endemic (green) settings, weak(light), moderate (medium) and strong (dark).

In endemic settings, rewiring movements increase the in- and out-degrees of the herds, i.e. the number of different herds they are connected to (Additional file 9). The increase is small but systematic, for every algorithm parameter value. In addition, the algorithm also affects the strongly connected components of the network in endemic settings. On the one hand, the algorithm reduces their number, all the more that the infection was strong (Figure 5). On the other hand, the size of the largest strongly connected component is increased in most, but not all simulations (64%, 67% and 80% of simulations in low, moderate and high endemic settings, respectively). It should be noted that the lesser impact of the algorithm on the network in epidemic settings can be explained by a number of rewiring events 25 times smaller on average than in endemic settings.

## Discussion

### Main results

The rewiring algorithm we developed for this study is able to reduce the extent of infections, in the absence of any other restriction measure and for a large panel of disease parameters (infection rate *β*, recovery rate *γ* or rate of return to susceptibility *δ*). However, the extent of the reduction varies between the different epidemiological settings considered. Indeed, infections are almost completely prevented with weak or moderate epidemic settings, while they still develop or persist for other settings, although not as much as without any rewiring. However, the decrease in the number of infected herds is not necessarily coupled with a decrease in the number of infected individuals. This result highlights the tendency of the algorithm to concentrate infected individuals in the already infected herds. The algorithm therefore performs a trade-off that is beneficial to the metapopulation as a whole—with fewer infected herds—but detrimental to the smaller number of already infected herds, in such a situation where movement rewiring is not combined with complementary on-farm measures to reduce within-herd infection prevalence. This is the case for the infections in an epidemic setting, in which the prevalence in the infected herds increases over the year. This is also the case for infections in endemic settings, in which new sensitive individuals could still be born or imported.

The sensitivity analysis on the infection-related outcomes reveals that the impact of the parameters of the algorithm is highly dependent on the epidemiological setting. Prohibiting the movements at risk, i.e., removing the movements that cannot be rewired and are delayed as much as possible, is mostly significant if the infection is not too strong and is just beginning. Only in these cases can the infection be fully contained by the rewiring. Increasing the maximal delay improves the performance of the algorithm in an endemic setting, for which the number of movements rewired is much larger than in epidemic settings. In those, delaying the movement to the next day increases substantially the opportunities for rewiring. The other two parameters are both related to the definition of the prevalence statuses used by the algorithm. A greater number of prevalence classes, which mainly impacts rewiring during strong infections, improves the separation of disease-free herds from the rest. Indeed, only for strong infection and *c* = 2 does the lowest prevalence class herds with no infected individual with herds with few infected individuals (Additional file 5), thus preventing the algorithm to efficiently protect disease-free ones. This result therefore supports the systematic inclusion of a class for herds with a zero prevalence in all cases. A longer update period between updates of the prevalence status makes the rewiring algorithm more error-prone, with a proportion of undetected movements at risk increasing with the time since the last update, at least up to 10 weeks. This result is visible for any epidemiological setting, suggesting that any increase in the frequency of update to the status of the herds should improve the effectiveness of the algorithm. Conversely, the results indicate that increasing the number of prevalence classes to more than two, or having a maximum delay greater than zero, improves the efficiency of the algorithm much more than further increases.

As expected, the impact of rewiring on the commercial movements network structure is limited, as it targeted a few movements only: less than 20% of the movements for endemic infections and less than 2% of them for epidemic infections. Nevertheless, rewiring tends to increase the overall connectedness of the herds during endemic infections. Indeed, the increase in degree and in size of the largest strong component indicates that the algorithm has connected herds that were originally not so. These metrics are generally correlated with higher expected epidemic risks [25, 43]. The use of such a rewiring method to manage actual bovine movements should take into account this potential increase in the risk of spreading other diseases. The algorithm could be extended to assess multiple diseases at once, but the additional constraints on rewiring would likely reduce its effectiveness.

The generality of the epidemiological model and of the algorithm used for this study makes the rewiring method we developed applicable to a much wider range of networks in animal and plant populations than just cattle, e.g. among seed exchange networks, which face similar infection risks [44, 45]. The SIRS model used allows for great flexibility in the type of disease considered, but the algorithm would also function with a disease-specific model, provided prevalences can be estimated from the epidemiological model used. Besides, while the need for controlled movements makes this method more relevant to agricultural systems, the spatial and temporal scales considered can also be adapted depending on the context. Indeed, the daily time-step and the region level were used here as they correspond to the BDNI data structure, but are not necessary for the algorithm to work. The usefulness of our rewiring method could therefore extend beyond cattle concerns, even though the effectiveness of the algorithm in other contexts remains to be tested.

### Limitations

Although the algorithm is tested on historical data from the BDNI for this study, it could also be intended to be used prospectively as part of decision-making tools. While it would be possible, actually using the algorithm would require overcoming some hurdles linked to its use in a real-life context.

First, one would have to ensure the quality of prevalence estimations. Indeed, our results show that the optimal functioning of the algorithm relies on getting accurate and frequent prevalence data from a large number of farms. While bulk milk-based sampling systems could be used for some diseases in cattle, e.g. with bovine viral diarrhoea [46, 47], this cannot be generalised to other diseases. Testing only a fraction of the animals in the herd can be envisioned to reduce costs, with an impact, albeit limited for the less severe diseases, on the assignment of herds to the correct prevalence status (Additional file 3). Besides, the test of our algorithm does not account for imperfect sensitivity or specificity of the tests used. While test specificity is generally high for cattle diseases [48–51], sensitivity can be low, thus limiting the number of infected animals detected and altering the observed prevalence. The impact of low sensitivity on erroneous herd status assignment is of the same order as that of testing only a fraction of the animals (Additional file 3). Their impact is likely mitigated by the use of prevalence classes, each of them spanning across a range of prevalence values, so that small inaccuracies in the prevalence estimation of a given herd does not necessarily change its assigned status. However, their impact also appears to be additive, so the decision to test fewer animals in each herd should for instance take test sensitivity into account in order to limit the overall error, particularly for the stronger diseases for which this error is generally greater.

Another way to reduce the sampling effort would be to focus on a subset of herds to monitor. Firstly, this sampling effort could take into account additional information available thanks to measures already in place. For instance, the status of some herds could be approximated through health accreditation schemes (e.g. [20]), with herds already identified as disease-free could be automatically assigned the lowest prevalence status for a given duration. Secondly, monitored herds could be selected based on their role in disease spread, notably through network metrics. Indeed, central herds in the movement network, i.e. those through which a large proportion of animal movements pass, are expected to play a larger role in the spread of infection [4, 52]. Hoscheit et al. [53] reviewed centrality measures taking into account the dynamic nature of the movement network, based on the BDNI. They found that the TempoRank index would for example be a good candidate for selecting a subset of herds to be specifically monitored and taken into account by the algorithm.

In this study, we use a network corresponding to commercial movements between every farm in Brittany (an administrative region of France) over the year 2014 to test the efficiency of the algorithm. On the one hand, the age of the data must be taken into account, as the structure of the current movement network on which the algorithm could potentially be used may have since changed, with potential implications on the algorithm performance. Variations in the network structure of the BDNI have been investigated over a 5-year period [5]. Results suggests that most network metrics remain largely stable over time, the main trend being a decrease in the number of herds in the network, likely because of acquisitions and mergers of farms. Although this change in the number of herds should be taken into account as it could modify the rewiring opportunities for the algorithm, the otherwise stability of the network indicates that our results should remain largely applicable to the current network. On the other hand, the spatial scale of the data must also be taken into account. The choice to consider a single region is notably motivated by computational limitations. Indeed, simulating a stochastic spread of the disease on a national scale over six years—five for the preliminary simulations and one for the main simulations—would have been considerably more costly, thus limiting the exploration of variations in the parameters of the SIRS model and the algorithm. Yet, this choice has additional implications that should be underlined.

Firstly, a substantial proportion of the movements involve herds outside of Brittany and are therefore not concerned by the rewiring. Indeed, 20% of all movements whose destination is in the metapopulation have an origin outside of it. In our simulations, these imports are assumed to not be movement at risk, i.e. that the prevalence status of their origin is never higher than that of their destination. This is not trivial, as it presumes that imports do not create greater infection risks than internal movements. In a real-life context, applying this rewiring method in a single region would therefore require an additional management of the risk associated with imports. Yet, extending its use nationally should mitigate this problem, as the proportion of imports is expected to be much lower at this scale. Secondly, every commercial movement between farms is considered to test the algorithm, regardless of breed or age, in order to have a large enough set of movements. Indeed, additional criteria, concerning for instance the breed of the animals, could be added easily by providing the algorithm with movements for individuals in each category separately. However, such criterion would reduce the rewiring possibilities of the algorithm and therefore its effectiveness. Again, the network of commercial movements at the national scale could be large enough to separate the movements by breed or consider only movements of specific breeds.

Finally, the implementation of such an algorithm would have to take into account farmers decision. Unless rewiring is enforced, the actual movements would result from sanitary concerns as well as other constraints, which would impact the effectiveness of the algorithm. Coupling it with a decision-making model could provide additional insight on this impact. In order to make it easier to use as part of such decision-making tools, the algorithm has been specifically designed to be able to include additional, different constraints.

## Conclusion

This study demonstrates the effectiveness of a rewiring method targeting specific movements to reduce infection risks. Our approach thus differs from that presented by Gates and Woolhouse [33], as it also aims at generating minimal changes in the structure of the movement network. This study also builds upon the results from Ezanno et al. [20], by confirming the effectiveness of this method beyond the case of a specific disease. Indeed, the algorithm presented by Ezanno et al. [20] and later by Biemans et al. [35], was developed specifically to address the control of bovine paratuberculosis, notably characterised by an endemic status and a low detection rate. To do so, they used a specific age-structured epidemiological model [54] and an algorithm calibrated to target this disease. This was also the case for instance of Mohr et al. [34], which specifically targeted foot-and-mouth disease. Conversely, the present study aims at assessing more comprehensively the effectiveness of the algorithm. It is tested for different epidemiological settings – both endemic and epidemic – using a non-specific epidemiological model, and for broad range of parameter values. This study is therefore complementary to the previous ones, by bringing a broader perspective on the impact of rewiring in animal movement network on infectious diseases in general.

## Supplementary Information

**Additional file 1. Description of the metapopulation epidemiological simulation model.**

**Additional file 2. Definition of the epidemiological settings.**

**Additional file 3. Errors in prevalence status assignment due to imperfect test sensitivity or incomplete herd sampling.**

**Additional file 4. Description of the rewiring algorithm in pseudo-code.**

**Additional file 5. Values of the boundaries between prevalence classes for the different epidemiological scenarios.**

**Additional file 6. Variations in the number of infected herds and infected individuals in the simulations.**

**Additional file 7. PCA as a first step of the multivariate sensitivity analysis.**

**Additional file 8. Distributions of the simulations on the first PCA axes depending on the algorithm parameters.**

**Additional file 9. Changes in in- and out-degree distributions in the movement network after rewiring.**

## Data Availability

The code for the algorithm, as well as additional scripts for formatting the data or running preliminary simulations and dummy test data, are freely available [55]. See the "Anonymous Access" section of the "SCM" tab for instructions on how to access the corresponding Git repository. The dataset used in the study is an extraction from the French national bovine identification database (BDNI), which is confidential, and therefore cannot be provided publicly.

## Abbreviation

BDNI: base de données nationale d’identification animale
