# Supplementary material for: Rewiring cattle movements to limit infection spread
Source: Vet Res. 2024 Sep 19;55:111. doi: 10.1186/s13567-024-01365-z (PMC11414270; doi:10.1186/s13567-024-01365-z)
Supplement: Supplementary file 1 — Additional file 1. Description of the metapopulation epidemiological simulation model. [file 13567_2024_1365_MOESM1_ESM.docx]

Additional file 1: Description of the metapopulation epidemiological simulation model

The simulation model used in the article describes the variations in the number of susceptible, infected and recovered individuals in each herd in the metapopulation at each date. It is stochastic and discrete in time and space. Each time-step (equal to a day) comprise a movement phase including the simulation of commercial exchanges and demographic events, and a transition phase corresponding to the simulation of infections, recoveries or returns to susceptibility.

The movement phase

As stated in the main text of the article, the term 'movement' is used here to refer to commercial exchanges as well as births and deaths of individuals. The five types of movements are always simulated in the following order: births, imports, internal movements, exports and deaths. This order has been chosen so that individuals are only added to the metapopulation before the movements and only removed afterwards. As there is no information about the actual order of movements within the same day, this ensures that there are enough individuals in the herds to perform the internal movements. Within each set of movements of the same type, the order used follows the list of movements provided by the database.

Each birth and import are simulated by adding an individual to the destination, each export and death by removing an individual from the origin. Internal movements are simulated by removing an individual from the origin and adding an individual to the destination.

The status of each moved individual is randomly drawn from a multinomial distribution with $n=1$ and probabilities of being susceptible $p_{S}\left( m \right)$, being infected $p_{I}\left( m \right)$, and being recovered $p_{R}\left( m \right)$:

$\left( M_{S},M_{I},M_{R} \right)\sim Multinomial\left( 1,p_{S}\left( m \right),p_{I}\left( m \right),p_{R}\left( m \right) \right)$.

Therefore, the status of the individual moved is susceptible if $M_{S}=1$, infected if $M_{I}=1$ and recovered if $M_{R}=1$. One individual is moved at a time, in order to avoid drawing more individuals of a given status than available in the origin.

The newborns are always susceptible, meaning that $p_{S}\left( m \right)=1$, while $p_{I}\left( m \right)=p_{R}\left( m \right)=0$ if $m$ is a birth. For internal movements, exports and deaths, the origin of the movement $O_{m}$ is a herd of the metapopulation, in which every animal has an equal chance of being chosen. Therefore, the probabilities for an animal of exiting herd $O_{m}$ at time $T_{m}$ are defined as follows:

$$p_{S}\left( m \right)=S_{O_{m}}\left( T_{m} \right)/N_{O_{m}}\left( T_{m} \right)$$

$$p_{I}\left( m \right)=I_{O_{m}}\left( T_{m} \right)/N_{O_{m}}\left( T_{m} \right)$$

$$p_{R}\left( m \right)=R_{O_{m}}\left( T_{m} \right)/N_{O_{m}}\left( T_{m} \right)$$

For imports, the proportions of susceptible, infected and recovered individuals of the origin are unknown. Instead, a phantom herd is created by pooling the herds of the metapopulation together, and used as an origin as described above. If there is no rewiring during the simulation, every herd is included into this phantom herd. If there is a rewiring event during the simulation, herd $h$ is included in the phantom herd created for an import $m$ only if its observed prevalence status is lower than or equal to the one of the destinations of the movement, i.e. if $V_{h}^{o}\left( T_{m} \right)\leq V_{h}^{D_{m}}\left( T_{m} \right)$.

| 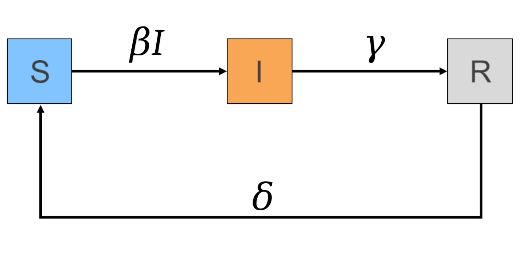 |
| --- |
| **Figure S1:** Functioning of the SIRS model, with susceptible(S), infected(I) and recovered (R) compartments, and the transition rates between the three states. |

The transition phase

The intra-herd phase is defined by a SIRS model with three parameters: the infection rate $\beta$, the recovery rate $\gamma$ and the rate of return to susceptibility $\delta$ (Figure S1). The transmission rate considered for this model is therefore density-dependent, meaning that the number of contacts between animals is expected to increase with the number of individuals, relative to the surface area available (Begon et al. [42], Álvarez et al. [38]). The surface areas of the different holdings considered being unknown here, they were not accounted for and considered constant.

The number of newly susceptible ($S_{h}^{'}\left( t \right)$), infected ($I_{h}^{'}\left( t \right)$) and recovered individuals ($R_{h}^{'}\left( t \right)$) in herd $h$ is drawn from binomial distributions:

$$S_{h}^{'}\left( t \right)\sim Binomial\left( R_{h}^{m}\left( t \right),\delta\right)$$

$$I_{h}^{'}\left( t \right)\sim Binomial\left( S_{h}^{m}\left( t \right),\beta I_{h}^{m}\left( t \right) \right)$$

$$R_{h}^{'}\left( t \right)\sim Binomial\left( I_{h}^{m}\left( t \right),\gamma\right)$$

with $S_{h}^{m}\left( t \right)$, $I_{h}^{m}\left( t \right)$ and $R_{h}^{m}\left( t \right)$ the numbers of susceptible, infected and recovered individuals in herd $h$ after the simulation of movements, respectively. The number of susceptible, infected and recovered individuals at $t+1$ is then computed as follows:

$$S_{h}\left( t+1 \right)=S_{h}^{m}\left( t \right)+S_{h}^{'}\left( t \right)-I_{h}^{'}\left( t \right)$$

$$I_{h}\left( t+1 \right)=I_{h}^{m}\left( t \right)+I_{h}^{'}\left( t \right)-R_{h}^{'}\left( t \right)$$

$$R_{h}\left( t+1 \right)=R_{h}^{m}\left( t \right)+R_{h}^{'}\left( t \right)-S_{h}^{'}\left( t \right)$$
