# Supplementary material for: Rewiring cattle movements to limit infection spread
Source: Vet Res. 2024 Sep 19;55:111. doi: 10.1186/s13567-024-01365-z (PMC11414270; doi:10.1186/s13567-024-01365-z)
Supplement: Supplementary file 2 — Additional file 2. Definition of the epidemiological settings. [file 13567_2024_1365_MOESM2_ESM.docx]

Additional file 2: Definition of the epidemiological settings

| 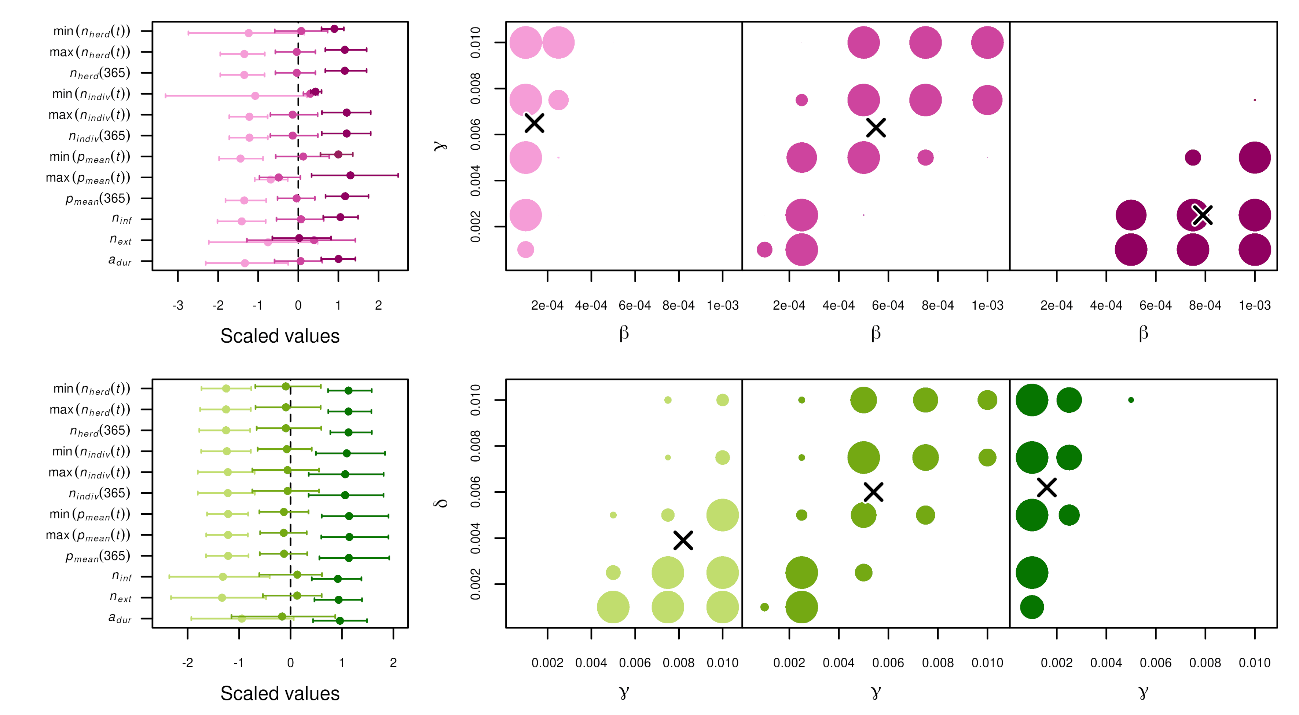 |
| --- |
| **Figure S2:** Clustering analyses for simulations with an endemic ($1^{st}$ row, magenta) or an epidemic infection type ($2^{nd}$ row, green). Left: Mean and 80% of the scaled variables for the simulations in the cluster of weak (light), moderate (medium) and strong (dark) setting. Right: distribution of the simulations in each cluster according to the values of $\beta$ and $\gamma$ (for epidemic settings) or the values of $\gamma$ and $\delta$ (for endemic settings). The size of the dots corresponds to the proportion of simulations in the settings. The crosses correspond to the values of the parameters averaged over all the simulations belonging to the cluster. |

The six infection settings presented in the main text of the article are defined according to two clustering analyses. These analyses are performed on sets of simulated infections in the metapopulation presented in the main text, comprising all cattle herds in Brittany between 01/01/2014 and 31/12/2014. The first clustering analysis is performed with an epidemic infection type, i.e. with an outbreak starting at the beginning of the simulation $t_{I}=t_{0}$. The second is performed with an endemic infection type, i.e. with an outbreak starting five years prior to the simulation $t_{I}=t_{0}-1825$ days. Three clusters are identified for each set of simulations, using the k-means method. Each set of simulations includes $5 \times5 \times5$ combinations of values of $\beta$ (${1.10}^{-4}$, ${2.5.10}^{-4}$, ${5.10}^{-4}$, $7.{5.10}^{-4}$, ${1.10}^{-3}$), $\gamma$ and $\delta$ (${1.10}^{-3}$, ${2.5.10}^{-3}$, ${5.10}^{-3}$, ${7.5.10}^{-3}$, ${1.10}^{-2}$ for each). The 125 combinations of parameter values are simulated 500 times each.

The infection-related outcomes considered are the one described in Table 1 of the main text of the article. Twelve variables are derived from these outcomes. The three outcomes computed once per simulation $n_{inf}$, $n_{ext}$ and $a_{dur}$ are considered as such. In addition, the maximum, minimum and final values of $n_{herd}\left( t \right)$, $n_{ind}\left( t \right)$ and $a_{prev}\left( t \right)$ are computed. They are respectively noted $max\left( u\left( t \right) \right)$, $min\left( u\left( t \right) \right)$ and $u\left( 365 \right)$ for outcome $u\left( t \right)$.

The 12 variables used for the clustering analysis are computed for each run of each setting and scaled, i.e. centred and divided by their standard deviation over all simulations. For both infection types (endemic and epidemic), the three clusters correspond to three levels of infection severity: weak, moderate and strong (Figure S2). However, the parameters driving the structuring of the clusters are different between the two infection types. For the epidemic infection type, clusters are mainly structured according to the $\beta$ value (positively correlated with the strength of the infection). The strong epidemic setting also differs from the others in having an overall lower value of $\gamma$ (Table S1). For the endemic infection type, clusters are mainly structured according to the $\gamma$ value (negatively correlated with the strength of the infection). In addition, the weak endemic setting has overall lower $\delta$ values.

| Epidemiological setting | | $\beta\left( \times{10}^{-3} \right)$ | $\gamma\left( \times{10}^{-3} \right)$ | $\delta\left( \times{10}^{-3} \right)$ |
| --- | --- | --- | --- | --- |
| Epidemic | Weak | 0.14 | 6.5 | 5.1 |
|  | Moderate | 0.55 | 6.3 | 5.5 |
|  | Strong | 0.79 | 2.5 | 5.5 |
| Endemic | Weak | 0.43 | 8.2 | 3.9 |
|  | Moderate | 0.55 | 5.4 | 6.0 |
|  | Strong | 0.58 | 1.6 | 6.2 |
| **Table S1:** Average values of $\beta$, $\gamma$ and $\delta$ for the simulations belonging to each cluster identified. | | | | |
