# Supplementary material for: Rewiring cattle movements to limit infection spread
Source: Vet Res. 2024 Sep 19;55:111. doi: 10.1186/s13567-024-01365-z (PMC11414270; doi:10.1186/s13567-024-01365-z)
Supplement: Supplementary file 3 — Additional file 3. Errors in prevalence status assignment due to imperfect test sensitivity or incomplete herd sampling. [file 13567_2024_1365_MOESM3_ESM.docx]

Additional file 3: Errors in prevalence status assignment due to imperfect test sensitivity or incomplete herd sampling

| 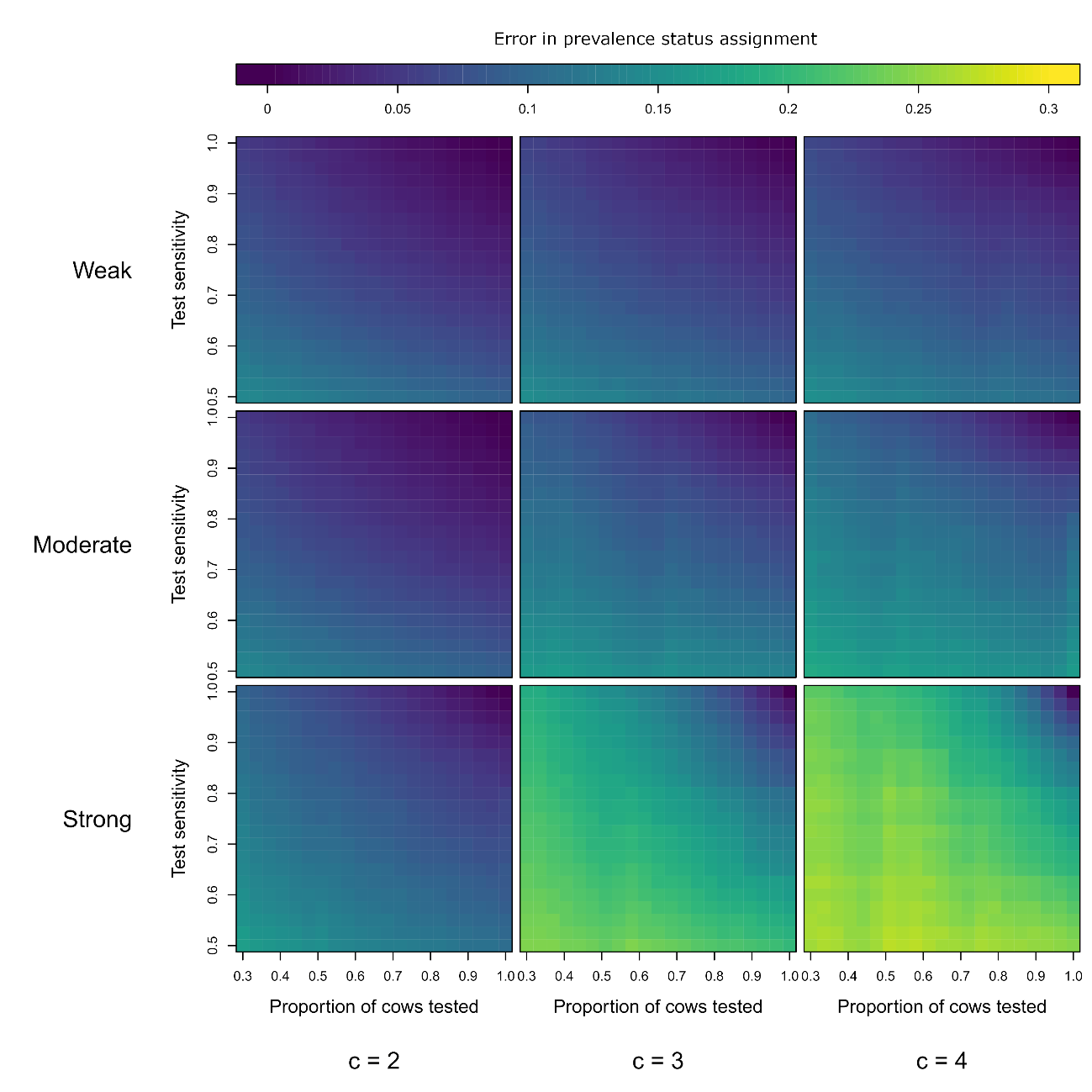 |
| --- |
| **Figure S3:** Error in prevalence status assignment for an endemic disease, as a function of the proportion of cows tested (from 30% to 100%) and the test sensitivity (from 0.5 to 1), for a weak (first row), moderate (second row) or strong disease (third row), and for $c=2$ (first column), $c=3$ (second column), $c=4$ (third column). |

The impacts of test sensitivity and of the proportion of animals tested are assessed using the distribution of herd prevalences observed at the start of simulations, i.e. $t=0$ for the endemic settings (weak, moderate and strong). Conversely to the epidemic setting, which simulates the early spread of the infection, the prevalences of the herds in the endemic setting are the result of five years of disease spread, with an outbreak at $t=-1825$ days. The metapopulation therefore displays a large range of prevalence values, with herds distributed equally among the different prevalence classes (see main text and Additional file 5), which increases the potential for wrong assignments of prevalence status because of imperfect test sensitivity or incomplete herd sampling. The endemic setting is therefore more suited as a worst-case scenario for detecting this kind of errors, compared to the epidemic one.

In the metapopulation, herd $h$ is characterised by its real prevalence $P_{h}\left( 0 \right)$ and its real status $V_{h}^{r}\left( 0 \right)$. Firstly, the testing of a fraction $a$ of the animals is simulated by randomly drawing $aN_{h}\left( 0 \right)$ animals (rounded to the nearest integer) from the $N_{h}\left( 0 \right)$ animals of herd $h$, with the number of infected animals among them noted $I_{h}^{s}\left( 0 \right)$. Secondly, the sensitivity of tests $b$ is taken into account by randomly drawing the number of positive individuals in a binomial distribution: $I_{h}^{i}\left( 0 \right)\sim Binomial\left( I_{h}^{s}\left( 0 \right),b \right)$. Finally, herd $h$ is assigned an imperfect prevalence status $V_{h}^{i}\left( 0 \right)$ according to the ratio between the number of positive individuals over the number of individuals tested. Error in status assignation is then computed as the proportion of herds in the metapopulation for which $V_{h}^{i}\left( 0 \right)\neq V_{h}^{r}\left( 0 \right)$.

The error in status assignation is computed for values of $a\in\left[ 0.3,1 \right]$ and values of $b\in\left[ 0.5,1 \right]$, for weak, moderate and strong endemic scenarios and for a number of prevalence classes $c$ of 2, 3, or 4 (Fig. S3). Results show that this error increases with either $a$ or $b$, with similar impacts of both values. The error also increases with the number of prevalence classes $c$, which is expected, as increasing the number of classes reduces the changes in prevalence necessary for a herd to be assigned another status. Yet, the maximum error never reaches 0.3, meaning that even in the worst case for which 30% of animals sampled and test sensitivity is of 0.5, at least 70% of herds are still assigned the correct prevalence status.

The impact clearly remains lower for weak and moderate diseases. For an intermediate case, with 65% of animals sampled in each herd and a sensitivity of 0.75, the error varies between 0.057 and 0.072 for a weak disease, and between 0.058 and 0.111 for a moderate one. For the worst case, with 30% of animals sampled and a sensitivity of 0.5, the errors vary respectively between 0.138 and 0.148 and between 0.139 and 0.181. For strong scenario and $c=2$, the error is slightly greater, with 0.091 for the intermediate case and 0.167 for the worst case. However, the error becomes substantially larger when considering strong diseases and a number of classes $c$ of 3 or 4. The errors raise respectively to 0.177 and 0.226 in the intermediate case and to 0.238 and 0.271 for the worst case.
