# Supplementary material for: Rewiring cattle movements to limit infection spread
Source: Vet Res. 2024 Sep 19;55:111. doi: 10.1186/s13567-024-01365-z (PMC11414270; doi:10.1186/s13567-024-01365-z)
Supplement: Supplementary file 4 — Additional file 4. Description of the rewiring algorithm in pseudo-code. [file 13567_2024_1365_MOESM4_ESM.docx]

Additional file 4: Description of the rewiring algorithm in pseudo-code

A given simulated time-step $t$ is separated into three successive steps: (1) the potential update of the observed herd statuses, (2) the rewiring of internal movements and (3) the simulation of the epidemiological model. This section presents the functioning of step 2 in pseudo-code.

Inputs fixed for the simulation __________________________________________________________

Given:

a number of estimated prevalence classes $c$ (integer $>$ 0)

a maximal delay $\Delta_{MAX}$ (integer $\geq$ 0)

a boolean indicating whether movements at risk were prohibited **PROHIB** (true or false)

Permutation of the origins _____________________________________________________________

For $c_{DR}$ from $1$ to ($c-1$), in ascending order:

For $c_{OR}$ from $c$ to ($c_{DR}+1$), in descending order:

For $c_{ON}$ from $1$ to $c_{DR}$, in ascending order:

For $c_{DN}$ from $c$ to $c_{OR}$, in descending order:

Set **lRisk** the list of all movements $mR$ such that $V_{O_{mR}}^{r}\left( t \right)=c_{OR}$ and $V_{D_{mR}}^{r}\left( t \right)=c_{DR}$

Set **lNorm** the list of all movements $mN$ such that $V_{O_{mN}}^{r}\left( t \right)=c_{ON}$ and $V_{D_{mN}}^{r}\left( t \right)=c_{DN}$

Set **minLen** the smallest value between the lengths of **lRisk** and of **lNorm**

If **minLen**$> 0$:

For $k$ between 1 and **minLen**:

Set **mRisk** the $k^{th}$ movement in **lRisk**

Set **mNorm** the $k^{th}$ movement in **lNorm**

Set **NewOR** the origin of **mNorm**

Set **NewON** the origin of **mRisk**

Change the origin of **mRisk** to **NewOR**

Change the origin of **mNorm** to **NewON**

Management of the remaining movements at risk__________________________________________

Set **lRemain** the list of movements $mE$ such that $V_{O_{mE}}^{r}\left( t \right)>V_{D_{mE}}^{r}\left( t \right)$

Set **lToDelay** the list of movements in **lRemain** $mD$ such that $T_{mD}<\left( T_{mD}^{*}+\Delta_{MAX} \right)$

Set **lProblem** the list of movements in **lRemain** $mP$ such that $T_{mP}=\left( T_{mP}^{*}+\Delta_{MAX} \right)$

For **mD** in **lToDelay**:

Increase $T_{\boldsymbol{mD}}$ by $1$

If **PROHIB**:

For movement **mP** in **lProblem**:

Set **newImport** as an import with $D_{\boldsymbol{newImport}}=D_{\boldsymbol{mP}}$ and $T_{\boldsymbol{newImport}}=t$

Set **newExport** as an export with $O_{\boldsymbol{newExport}}=O_{\boldsymbol{mP}}$ and $T_{\boldsymbol{newExport}}=t$

Replace **mP** by **newImport** and **newExport**
