# Supplementary material for: Rewiring cattle movements to limit infection spread
Source: Vet Res. 2024 Sep 19;55:111. doi: 10.1186/s13567-024-01365-z (PMC11414270; doi:10.1186/s13567-024-01365-z)
Supplement: Supplementary file 5 — Additional file 5. Values of the boundaries between prevalence classes for the different epidemiological scenarios. [file 13567_2024_1365_MOESM5_ESM.docx]

Additional file 5: Values of the boundaries between prevalence classes for the different epidemiological scenarios

Each prevalence class is defined by two boundaries, such that class $i$ includes herds whose prevalence is between a lower boundary $b_{i}$ and an upper boundary $b_{i+1}$. The lowest boundary ($b_{1}$) is always 0 and the highest boundary ($b_{c+1}$) is always 1. The other boundaries correspond to quantiles of the distribution of prevalence observed at the end of preliminary simulations without any rewiring, performed over five years between the 01/01/2009 and the 31/12/2014. Therefore, they are susceptible to vary, especially depending on the epidemiological scenario considered. Fig. S4 presents the values of these boundaries for 500 preliminary simulations for each epidemiological scenario.

Results show that the first class always includes the non-infected herds only ($b_{2}$ = 0), except for $c$ = 2 for the strong epidemic and endemic scenarios (Figure S4A), for which the $b_{2}$ is respectively 0.32 and 0.50, on average. For $c$ = 3, the value of ($b_{3}$) increases with the severity of the epidemiological scenario (weak, moderate strong), but it is comparable between epidemic and endemic scenarios of the same severity, although slightly higher for the endemic one (Figure S4B). The results are similar for $c$ = 4, for which $b_{4}>b_{3}$ (by definition), and those values increase greatly with the severity of the epidemiological scenario, but remain comparable between epidemic and endemic scenarios of the same severity.

| 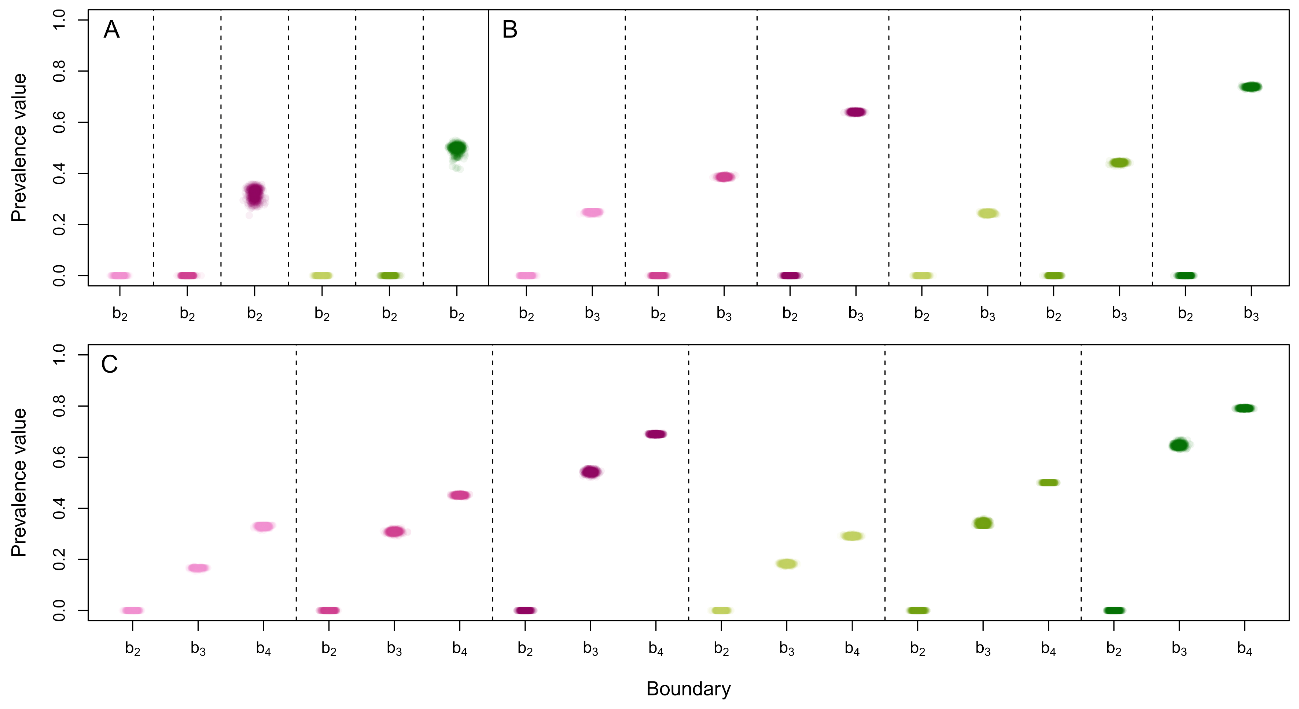 |
| --- |
| **Figure S4:** Values of the prevalence boundaries for epidemic (magenta) or endemic (green) diseases, weak (light), moderate (medium) or strong (dark), for $c$ = 2 (A), for $c$ = 3 (B) or for $c$ = 4 (C). Values of $b_{1}$ and $b_{c+1}$ are not represented, as $b_{1}$ = 0 and $b_{c+1}$ = 1 by definition. |
