# Supplementary material for: Rewiring cattle movements to limit infection spread
Source: Vet Res. 2024 Sep 19;55:111. doi: 10.1186/s13567-024-01365-z (PMC11414270; doi:10.1186/s13567-024-01365-z)
Supplement: Supplementary file 6 — Additional file 6. Variations in the number of infected herds and infected individuals in the simulations. [file 13567_2024_1365_MOESM6_ESM.docx]

Additional file 6: Variations in the number of infected herds and infected individuals in the simulations

| 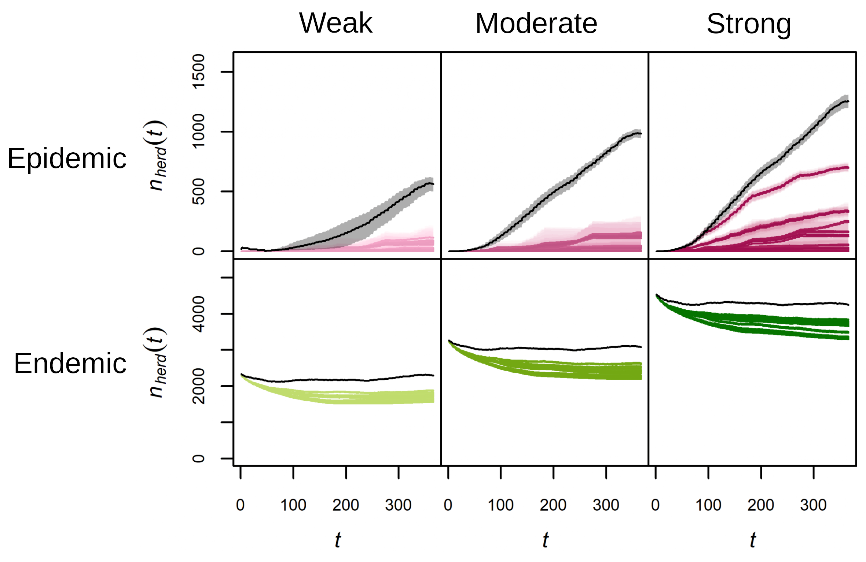 |
| --- |
| **Figure S5:** Number of infected herds $n_{herd}\left( t \right)$ as a function of time ($t$, in days), for simulations with (colour) or without rewiring (black), in epidemic ($1^{st}$ row, magenta) or endemic ($2^{nd}$ row, green) settings, weak (light), moderate (medium) or strong (dark). Each scenario (algorithm parameter combination) is represented by its mean over the repetitions (solid line) and an interval of 80% of simulations (envelope). |
| 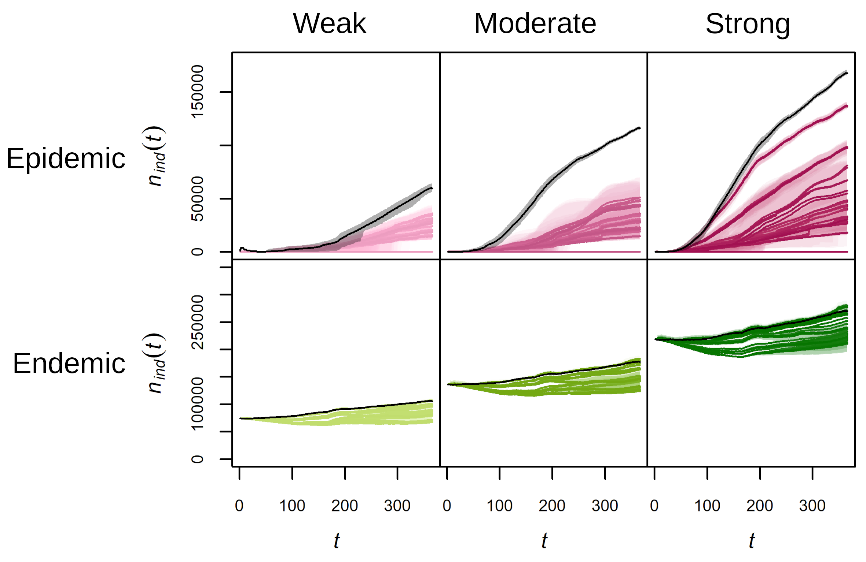 |
| **Figure S6:** Number of infected individuals $n_{ind}\left( t \right)$ as a function of time ($t$, in days), for simulations with (colour) or without rewiring (black), in epidemic ($1^{st}$ row, magenta) or endemic ($2^{nd}$ row, green) settings, weak (light), moderate (medium) or strong (dark). Each scenario (algorithm parameter combination) is represented by its mean over the repetitions (solid line) and an interval of 80% of simulations (envelope). |

The results presented here show the variation in the number of infected herds $n_{herd}\left( t \right)$ and infected individuals $n_{ind}\left( t \right)$ during the simulations, with and without rewiring, for each epidemiological setting. Simulations with rewiring are performed for scenario (i.e. combination of algorithm parameters) presented in the main text of the article. The values of $n_{herd}\left( t \right)$ (Figure S5) show a substantial impact of the algorithm in reducing infection, regardless of the scenario. The algorithm slows down the increase in number of infected herds, even reaching $n_{herd}\left( t \right)=0$ for some scenarios in epidemic settings. Besides, the algorithm also reduces $n_{herd}\left( t \right)$ in endemic settings, while it remains broadly constant during simulations without rewiring.

The values of $n_{ind}\left( t \right)$ (Figure S6) show a similar, albeit smaller, improvement brought about by the rewiring. The number of infected individuals is also lower for all scenarios in epidemic settings, although the difference is not as pronounced. However, this is not always the case in endemic settings, meaning that, for some scenarios, the algorithm only concentrates infected cattle into a smaller number of herds, without reducing the total number of infected animals.
