# Supplementary material for: Rewiring cattle movements to limit infection spread
Source: Vet Res. 2024 Sep 19;55:111. doi: 10.1186/s13567-024-01365-z (PMC11414270; doi:10.1186/s13567-024-01365-z)
Supplement: Supplementary file 7 — Additional file 7. PCA as a first step of the multivariate sensitivity analysis. [file 13567_2024_1365_MOESM7_ESM.docx]

Additional file 7: PCA as a first step of the multivariate sensitivity analysis

| 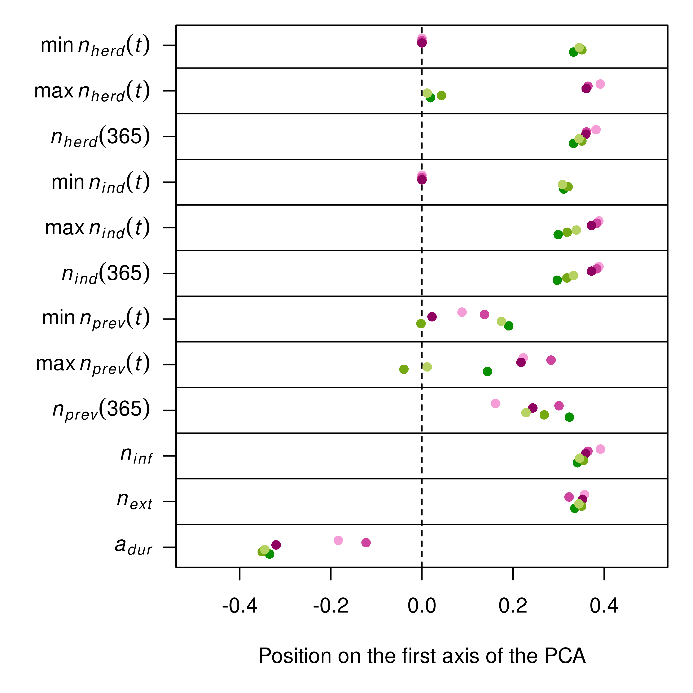 |
| --- |
| **Figure S7:** Position of the twelve infection-related outputs on the first principal component of the PCA prior to the sensitivity analysis, for the weak (light), moderate (medium) and strong (dark) epidemic (magenta) and endemic (green) settings. |

The multivariate sensitivity analysis following Lamboni et al. [41] includes a preliminary principal component analysis (PCA) on the outputs, which are described in the main text of the article. While the analysis takes all the components of the PCA into account, the first principal component (FPC) is specifically investigated in the following. Its inertia is presented in Table S2 for each epidemiological scenario, and shows that it systematically captures a majority -- but not all -- of the variability in the data analysed.

|  |  | Inertia of the $1^{st}$ component of the PCA |
| --- | --- | --- |
| Epidemic | Weak | 55.56% |
|  | Moderate | 63.87% |
|  | Strong | 70.73% |
| Endemic | Weak | 68.58% |
|  | Moderate | 64.08% |
|  | Strong | 68.82% |
| **Table S2:** Inertia of the first principal component of the PCA prior to the sensitivity analyses. | | |

The correlation of the FPC with each output is very similar for each epidemiological setting (Fig. S7). The correlations observed suggest that this FPC describes well the extent of the simulated infection. Indeed, it is clearly positively correlated with outcomes corresponding to more extensive infections ($n_{herd}$, $n_{indiv}$, $n_{inf}$). Exceptions include $max\left( n_{herd}\left( t \right) \right)$ in endemic settings and $min\left( n_{herd}\left( t \right) \right)$ and $min\left( n_{herd}\left( t \right) \right)$ in epidemic settings, which are never correlated with the FPC. This is expected, as the number of infected herds always decreases with rewiring in endemic settings, and the number of infected herds and individuals always increase in epidemic settings (see Additional file 4). These values therefore correspond to those at $t = 0$, which are the same (or close to) across all simulations. The correlation between the FPC and the outcomes concerning prevalence ($n_{prev}$) is not as clear, but also overall positive.

However, the FPC is positively correlated to $n_{ext}$ and negatively correlated to $a_{dur}$, which is unexpected. This can be explained by their strong link with $n_{inf}$, the total number of infections of herds (Table S3). First, $n_{ext}$ is always strongly positively correlated with $n_{inf}$, as the extinction of the infection in a herd can only occur after the infection of this herd. Secondly, $a_{dur}$ is negatively correlated with $n_{inf}$, meaning that short-lived infections are more likely if many herds are infected. This could be the result of the algorithm: by concentrating infected individuals in fewer herds, the algorithm creates few, long-lived infections, compared to the infections observed when the algorithm was less effective.

| Epidemiological setting | | $cor(n_{inf},n_{ext})$ | $cor(n_{inf},a_{dur})$ |
| --- | --- | --- | --- |
| Epidemic | Weak | 0.85 | -0.44 |
|  | Moderate | 0.83 | -0.32 |
|  | Strong | 0.98 | -0.78 |
| Endemic | Weak | 0.99 | -0.99 |
|  | Moderate | 0.98 | -0.98 |
|  | Strong | 0.98 | -0.97 |
| **Table S3:** Pearson's correlations between $n_{inf}$ and $n_{ext}$ or $a_{dur}$ (scaled values) for each epidemiological scenario. | | | |
