# Supplementary material for: Rewiring cattle movements to limit infection spread
Source: Vet Res. 2024 Sep 19;55:111. doi: 10.1186/s13567-024-01365-z (PMC11414270; doi:10.1186/s13567-024-01365-z)
Supplement: Supplementary file 8 — Additional file 8. Distributions of the simulations on the first PCA axes depending on the algorithm parameters. [file 13567_2024_1365_MOESM8_ESM.docx]

Additional file 8: Distributions of the simulations on the first PCA
axes depending on the algorithm parameters

| 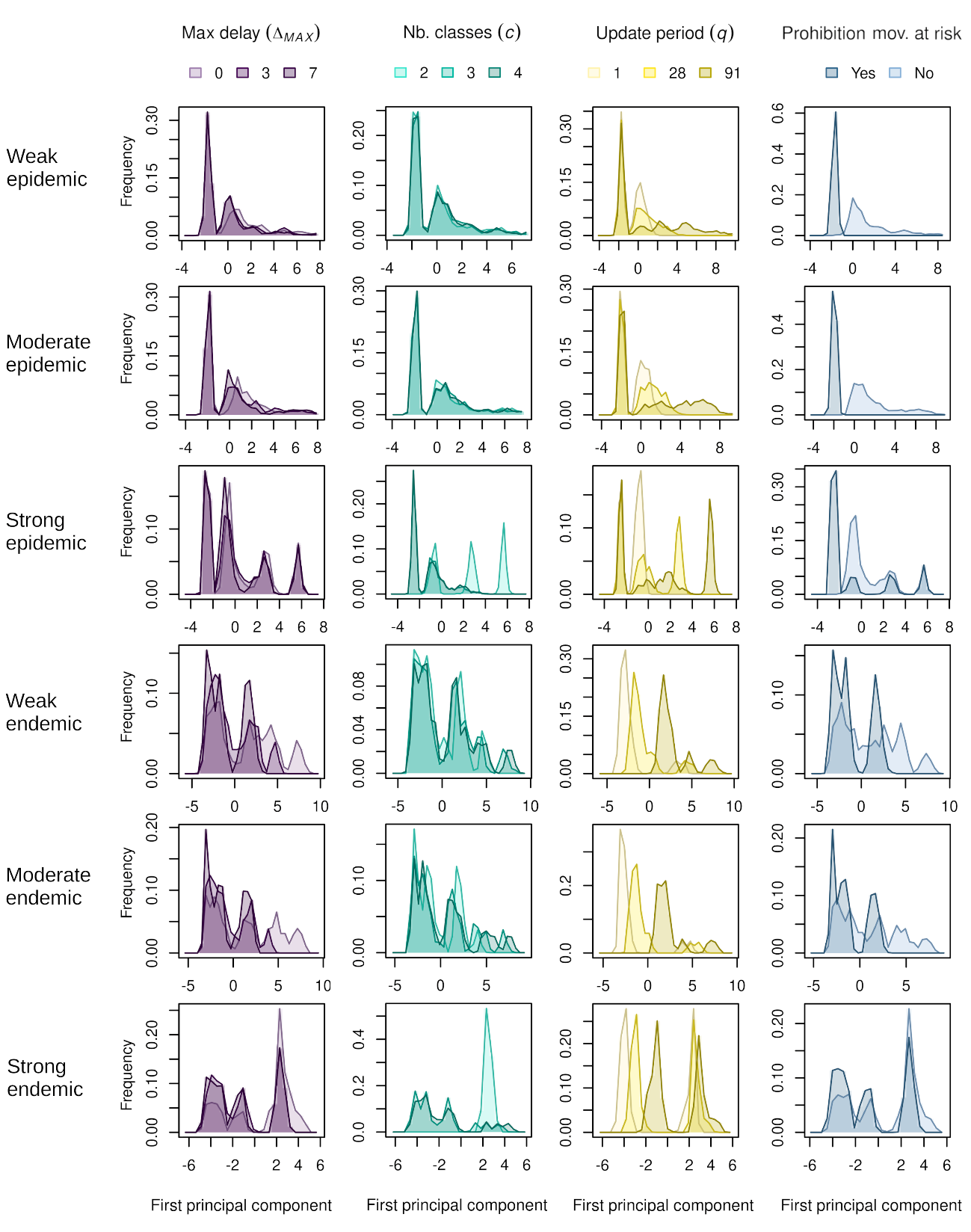 |
| --- |
| **Figure S8:** Distribution of the simulations on the first principal component of the PCA performed as a first step of the sensitivity analysis, for the six epidemiological settings (rows), according to their algorithm parameter values (columns). The simulations are divided according to their maximal delay (3 shades of purple), their number of prevalence classes (3 shades of cyan), herd status update period (3 shades of yellow) and prohibition of movements at risk (2 shades of blue). |
