# Supplementary material for: Rewiring cattle movements to limit infection spread
Source: Vet Res. 2024 Sep 19;55:111. doi: 10.1186/s13567-024-01365-z (PMC11414270; doi:10.1186/s13567-024-01365-z)
Supplement: Supplementary file 9 — Additional file 9. Changes in in- and out-degree distributions in the movement network after rewiring. [file 13567_2024_1365_MOESM9_ESM.docx]

Additional file 9: Changes in in- and out-degree distributions in the movement network after rewiring

| 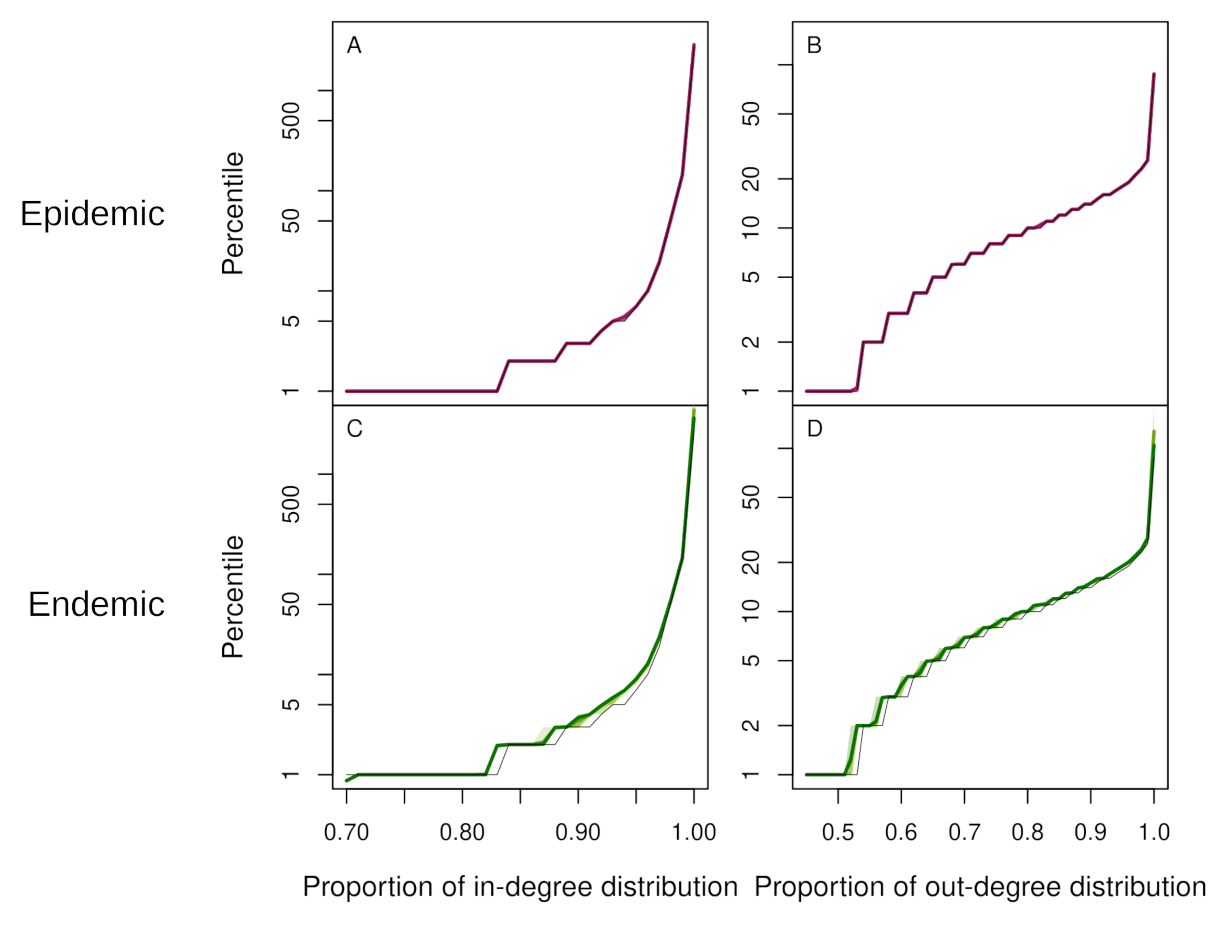 |
| --- |
| **Figure S9:** Percentiles (in log-scale) of the in-degree $ind_{h}$ ($1^{\mathrm{st}}$ column) and out-degree $outd_{h}$ ($2^{\mathrm{nd}}$ column) distributions after rewiring in epidemic ($1^{\mathrm{st}}$ row, magenta) and endemic settings ($2^{\mathrm{nd}}$ row, green), compared the distribution those of the original network (black). Null percentiles are not displayed. Each scenario (combination of algorithm parameters) is represented by its average (solid line) and an interval with 80% of simulations (envelope). Given the very low variance between simulations within a same epidemiologic scenario, envelopes are not represented if all 80% of simulations have the same percentile value. |

To assess the impact of the algorithm on the in- and out-degrees of the movement network, the distributions of $ind_{h}$ and $outd_{h}$ are recorded and their respective percentiles computed for each simulation. The distribution of percentiles for all simulations with the same epidemiological setting (epidemic or endemic) are compared to those of the original, non-rewired network. By definition, $x\%$ of herds have a degree lower or equal to the $x^{th}$ percentile value. In the original dataset, respectively 70% and 45% of the herds don't buy and sell livestock during the year. These proportions could only be modified by the algorithm through the replacement of internal movements by imports and exports, which would only decrease the degrees of the herds in question. Therefore, the following focuses on the distributions between the ${70}^{\mathrm{th}}$ and ${100}^{\mathrm{th}}$ percentile of $ind_{h}$ and between the ${45}^{\mathrm{th}}$ and ${100}^{\mathrm{th}}$ percentile of $outd_{h}$.

There is no strong difference between the degrees before and after rewiring in epidemic settings. Indeed, Figures S9A and S9B show that at least 80% of the rewired networks in epidemic settings had degree distributions almost identical to the distribution of the original network, regardless of algorithm parameter values. In endemic settings however, the percentile values are overall higher after rewiring. Indeed, the percentiles of $ind_{h}$ distribution above the ${83}^{\mathrm{th}}$ are on average 16% higher in rewired network than in the original one (Figure S9). Similarly, the percentiles of $outd_{h}$ distribution above the ${52}^{\mathrm{th}}$ are on average 9% higher (S9D). These results indicate a slight increase of the in- and out-degrees of the herds because of rewiring. This increase is evenly distributed across all herd degree levels, indicating that rewiring affects all herds regardless of degree. Besides, Figures S9C and S9D show that the increase is also observed across all simulations: the envelopes including 80% of the simulations being steadily over the percentile values for the original network indicate that at least 90% of the simulations experience the overall increase in in- and out-degree described above. Even though the increase remains small, it is therefore a consistent impact of the algorithm in endemic settings.
